# Association between Anemia and Auditory Threshold Shifts in the US Population: National Health and Nutrition Examination Survey

**DOI:** 10.3390/ijerph17113916

**Published:** 2020-06-01

**Authors:** Jui-Hu Shih, I-Hsun Li, Ke-Ting Pan, Chih-Hung Wang, Hsin-Chien Chen, Li-Yun Fann, Jen-Ho Tseng, Li-Ting Kao

**Affiliations:** 1 Department of Pharmacy Practice, Tri-Service General Hospital, Taipei 11490, Taiwan; jtlovehl@gmail.com (J.-H.S.); lhs01077@gmail.com (I.-H.L.); 2 School of Pharmacy, National Defense Medical Center, Taipei 11490, Taiwan; 3 Department of Pharmacology, National Defense Medical Center, Taipei 11490, Taiwan; 4 Institute of Environmental Design and Engineering, Bartlett School, University College London, London WC1E, UK; pankt930@gmail.com; 5 Graduate Institute of Aerospace and Undersea Medicine, National Defense Medical Center, Taipei 11490, Taiwan; 6 Graduate Institute of Medical Sciences, National Defense Medical Center, Taipei 11490, Taiwan; chw@ms3.hinet.net; 7 Department of Otorhinolaryngology, Taichung Armed Forces General Hospital, Taichung 41152, Taiwan; 8 Department of Otorhinolaryngology-Head and Neck Surgery, Tri-Service General Hospital, Taipei 11490, Taiwan; acolufreia@yahoo.com.tw; 9 Department of Nursing, Taipei City Hospital, Taipei 106, Taiwan; fanly99@gmail.com; 10 Department of Biology and Anatomy, National Defense Medical Center, Taipei 11490, Taiwan; 11 Department of Neurosurgery, Taipei City Hospital, Taipei 106, Taiwan; DAL87@tpech.gov.tw; 12 Graduate Institute of Life Sciences, National Defense Medical Center, Taipei 11490, Taiwan; 13 School of Public Health, National Defense Medical Center, Taipei 11490, Taiwan

**Keywords:** anemia, hearing loss, auditory threshold shifts, pure tone average

## Abstract

Existing evidence indicates that both iron deficiency anemia and sickle cell anemia have been previously associated with hearing loss. However, human data investigating the association between anemia and auditory threshold shifts at different frequencies in the adolescent, adult and elderly population are extremely limited to date. Therefore, this cross-sectional study used the dataset from the US National Health and Nutrition Examination Survey from 2005 to 2012 to explore differences in low- or high-frequency hearing thresholds and hearing loss prevalence between participants with and without anemia. A total of 918 patients with anemia and 8213 without anemia were included. Results indicated that low- and high-frequency pure tone average were significantly higher in patients with anemia than that in those without anemia in the elderly, but not in adult or adolescent population. In addition, the prevalence of low-frequency hearing loss but not high-frequency hearing loss was also higher in patients with anemia than in those without anemia in the elderly population. After adjusting various confounders, multiple regression models still indicated that patients with anemia tended to have larger threshold shift. In conclusion, anemia was associated with auditory threshold shifts in the elderly population, especially those vulnerable to low-frequency hearing loss.

## 1. Introduction

Approximately 466 million people worldwide have disabling hearing loss in 2018 [1], and unaddressed hearing loss poses an annual global cost of US$ 750–790 billion [2]. In the United States, the number of adults with hearing loss (pure tone average (PTA), >25 dB) is estimated to gradually increase from 44.1 million in 2020 to 73.5 million by 2060 [3]. Hearing loss is a sensory impairment caused by multiple factors including gene mutations, environmental noise, viral infection, autoimmune disease, labyrinthine membrane rupture, vascular events (e.g., vascular disease/alteration of microcirculation, vascular disease associated with mitochondriopathy, vertebrobasilar insufficiency, red blood cell deformability, sickle cell disease and cardiopulmonary bypass), and blood disorders [4]. Anemia is the most common blood disorder and also remains the major global public health problems. Existing evidence indicated that both iron deficiency anemia (IDA) and sickle cell anemia (SCA) have been previously associated with hearing loss [5,6].

In a Taiwan population-based study, Chung et al. found that the odds ratio (OR) for having a previous IDA diagnosis among patients aged ≥18 years with sudden sensorineural hearing loss was 1.34 (95% confidence interval (CI), 1.11–1.61), which was most pronounced among those aged ≤44 years compared with controls (OR, 1.91; 95% CI, 1.35–2.72) [7]. US cohort studies by Schieffer et al. also showed that children and adolescents with IDA have an increased risk of developing sensorineural hearing loss (OR, 3.67; 95% CI, 1.60–7.30); increased odds of sensorineural hearing loss (OR, 1.82; 95% CI, 1.18–2.66) but conductive hearing loss (OR, 1.51; 95% CI, 0.54–3.28) among adults with IDA [8,9]. In addition, a prospective case-control study by Aderibigbe et al. indicated that the average hearing thresholds of patients with SCA aged 16–48 years were significantly higher than controls aged 15–39 years in both right and left ears (right hearing thresholds: 16.7 ± 13.0 vs. 11.1 ± 9.1 dB; left hearing thresholds: 12.8 ± 9.4 vs. 9.6 ± 5.8 dB) [10].

Although IDA and SCA mechanisms that lead to hearing loss differ, hemoglobin may play a common and critical role in the deterioration of hearing function. In addition, typical changes in age-related hearing loss usually start with a hearing loss on high frequencies [11], and there are rare reports of low-frequency hearing loss occurring in old age. To date, human data investigating the association between anemia and auditory threshold shifts at different frequencies in the adolescent, adult and elderly population are extremely limited. Therefore, we use the dataset from the National Health and Nutrition Examination Survey (NHANES) to explore differences in low- or high-frequency hearing thresholds and hearing loss prevalence between participants with and without anemia.

## 2. Materials and Methods

### 2.1. Database

The data used in this cross-sectional study were derived from the NHANES in the United States (https://wwwn.cdc.gov/nchs/nhanes/). This survey includes information about questionnaires, demographic data, laboratory tests, and physical examinations, among others. All the study participants in the NHANES are sampled from the residents in the United States. Its research protocols were approved by the Research Ethics Review Board of National Center for Health Statistics. All participants provided written informed consents. Because we only used de-identified secondary data from NHANES, this study was exempted from full review by the Institutional Review Board.

### 2.2. Study Participants Selection and Anemia Definition

This study attempted to identify the association between anemia and auditory threshold shifts. Therefore, study participants aged 12 years and older were only limited to 2005–2012 NHANES individuals who underwent both audiometric examinations and with complete blood count results. Those with incomplete audiometric exams or missing laboratory tests for hemoglobin were excluded from this study. A total of 9131 US participants were recruited in this cross-sectional study. We then classified the study participants into two groups: patients with and without anemia. Anemia was defined based on the World Health Organization recommendations. Male participants with the serum hemoglobin level of <13 g/dL or female participants with the serum hemoglobin level of <12 g/dL were identified as patients with anemia [12]. Finally, 918 patients with anemia and 8213 without anemia were included in this study.

### 2.3. Audiometric Measures

According to the NHANES protocols, audiometric examinations excluded participants who could not tolerate headphones because of ear pain at the exam time. Those using hearing aids (not able to remove them) during the test were also excluded. All audiometry examinations were carried out by professionally trained audiologists from the National Institute for Occupational Safety & Health using the interacoustics model AD226 audiometer (with standard TDH-49 headphones and Etymotic EarTone 3A insert earphones). The hearing threshold test in this study was conducted on both right and left ears at 0.5, 1, 2, 3, 4, 6, and 8 kHz across an intensity range of −10 to 120 decibels (dB). Full audiometric protocols and procedures are available online.

### 2.4. Auditory Threshold Shifts and Hearing Loss Definition

In the relevant analyses of this study, PTA thresholds of hearing at 0.5, 1, and 2 kHz were identified as the low-frequency PTA (low PTA) and PTA thresholds of hearing at 3, 4, and 6 kHz were identified as the high-frequency PTA (high PTA) [13]. Additionally, this cross-sectional study identified patients with PTA at 0.5, 1, and 2 kHz thresholds of ≥15 dB in either ear as those with low-frequency hearing loss (LFHL). Patients who met the criteria for PTA at 3, 4, and 6 kHz thresholds of ≥15 dB in whichever ear were defined as the high-frequency hearing loss (HFHL). Furthermore, patients who experienced any hearing loss of ≥15 dB (including HFHL or LFHL) were identified as those with overall hearing loss (HL).

### 2.5. Covariate Measurement

To eliminate the potential effects of some confounders and investigate the actual relationship between anemia and HL, participants’ age group, sex, race, hypertension, diabetes, coronary heart disease, heart failure, and stroke were considered in the regression models. In this study, the race was categorized as non-Hispanic white, non-Hispanic black, or others (including Mexican American, other Hispanic, and other races). In addition, participants were considered to have medical history of hypertension, coronary heart disease, heart failure, and stroke if participants self-reported to have medical history about these diseases according to their physicians. Moreover, patients self-reported to have diabetes by physicians or other health professionals or receive diabetic medications were identified as diabetes cases.

### 2.6. Statistical Analysis

All analyses were performed with the SAS system (SAS System for Windows, ver. 9.4, SAS Institute Inc., Cary, NC, USA). This study used the chi-squared tests to explore differences in age group, sex, race, hypertension, diabetes, coronary heart disease, heart failure, and stroke, LFHL, HFHL, HL, between participants with and without anemia. Independent t-tests were further conducted to compare differences in low PTA and high PTA between participants with and without anemia. Additionally, regression models were carried out to estimate the effects of anemia on PTA thresholds of hearing. A two-sided *p*-value of 0.05 was used to determine the statistical significance of this study.

## 3. Results

This study consisted of 918 participants with anemia and 8213 without anemia. Table 1 shows the demographic characteristics and comorbidities of participants with anemia and controls without anemia. Relevant findings indicated that there were significant differences in age group (*p* < 0.001), sex (*p* < 0.001), and race (*p* < 0.001) between the two groups. In addition, the prevalence of hypertension (*p* < 0.001), diabetes (*p* < 0.001), coronary heart disease (*p* < 0.001), heart failure (*p* < 0.001), and stroke (*p* < 0.001) between participants with anemia and controls without anemia was significantly different.

Table 2 displays the high PTA, low PTA, and prevalence of LFHL, HFHL, and HL in participants with and without anemia. Results showed that participants with anemia had significantly higher low PTA (right ear: 15.04 dB vs. 11.64 dB; left ear: 15.57 dB vs. 11.56 dB) and high PTA (right ear: 23.82 dB vs. 18.55 dB; left ear: 24.71 dB vs. 19.51 dB) in both ears compared to participants without anemia. Moreover, the HL was found in 64.16% of participants with anemia and in 54.60% of controls without anemia (*p* < 0.001). LFHL was observed in 42.59% of participants with anemia and in 32.66% of controls without anemia (*p* < 0.001). Furthermore, the HFHL was found in 61.44% of participants with anemia and in 51.88% of controls without anemia (*p* < 0.001).

Because age is an important factor in the development of HL, Table 3 further displays the high PTA, low PTA, and prevalence of LFHL, HFHL, HL in participants with and without anemia according to the age group. Independent t-tests presented that participants with anemia had a significantly greater low PTA and high PTA than those without anemia among the elderly population (aged ≥ 60 years) in both ears. The prevalence of LFHL was also higher in participants with anemia than that in those without anemia. However, no significant difference in low PTA and high PTA was observed between patients with and without anemia among adult and adolescent population.

To reduce the potential effects of confounders, the regression models were used to investigate the relationships between PTA and anemia according to different age groups (Table 4). After adjusting various confounders, multiple regression models still indicated that patients with anemia tended to have higher hearing thresholds. Anemia was also significantly positively associated with the hearing thresholds, including both high PTA and low PTA, in the overall and elderly population. β coefficients of the high PTA comparing participants with anemia to controls without anemia were 2.35 for the right ear (*p* < 0.001) and 2.33 for the left ear (*p* < 0.001), and β coefficients of the low PTA were 1.53 for the right ear (*p* < 0.001) and 2.25 for the left ear (*p* < 0.001) in the overall population. Additionally, β coefficients of the high PTA comparing participants with anemia to controls without anemia were 3.76 for the right ear (*p* = 0.002), and 4.03 for the left ear (*p* = 0.001) and β coefficients of the low PTA were 3.55 for the right ear (*p* < 0.001) and 4.64 for the left ear (*p* < 0.001) in the elderly population. Additionally, this study performed the regression models to investigate the relationships between PTA and anemia according to different sexes and ethnic groups (Supplementary Table S1). After adjustments, anemia was positively associated with the hearing thresholds, including both high PTA and low PTA, in women, men, non-Hispanic white, and non-Hispanic black populations.

## 4. Discussion

In this study, anemia was found to be associated with auditory threshold shifts, which can be used to explain the higher prevalence of LFHL in elderly participants with anemia. To date, the mechanism of anemia-associated HL remains unclear and non-conclusive, although some plausible mechanisms were proposed. In the cochlear, intricate vasculature provides oxygen and nutrients needed for the stria vascularis of the cochlear duct to maintain the ionic composition of the endolymph and the endocochlear potential [14,15]. As anemia could decrease oxygen delivery in the labyrinthine arterial blood due to reduced hemoglobin concentration, blood oxygen supply to the cochlear is highly sensitive to ischemic damage.

Previous studies indicated that IDA is a potential risk factor for ischemic stroke [16], and patients with vascular disease have a higher risk for developing sudden sensorineural HL [7,17,18,19,20]. Iron is not only an essential component of hemoglobin in the red blood cells for tissue oxygen delivery but also a cofactor in neurotransmitter metabolism, DNA synthesis, and nerve myelination [21,22,23,24]. Neurological disorders might be associated with sensorineural HL. A systematic review and meta-analysis showed that compared with individuals without IDA, the age-specific OR of sensorineural HL was higher for children (3.67, 95% CI 1.72–7.84) than for adults (1.36, 95% CI 1.15–1.61; *p* = 0.27) [5]. However, participants with anemia have higher values of low and high PTA than those without anemia in the elderly but not the adult or adolescent population in this study (Table 3). We found that adolescent population with anemia are not apparent with low PTA threshold shift of 0.42–0.54 dB and high PTA threshold shift of 0.35–0.76 dB; low PTA threshold shift of 0.69–1.34 dB but high PTA negative threshold shift of 0.38–1.19 dB in the adult population. Elderly participants with anemia have remarkably low PTA threshold shift of 2.94–4.24 dB and high PTA threshold shift of 2.98–3.54 dB. Therefore, it is speculated that the biological interaction of aging and anemia would be a chronic progressive contributing factor to HL in the elderly population. In contrast, it may be different from IDA that has been regarded as a deteriorating factor of sudden sensorineural HL among adults aged <44 years [7].

Presbycusis, or age-related HL, is a common disorder characterized by symmetrical progressive loss of high-frequency hearing over the years. The World Health Organization estimates that >500 million people aged >60 years worldwide will suffer significant impairment from presbycusis by 2025 [25]. In this study, although elderly people with anemia have higher high PTA threshold values, similar prevalence of HFHL was found between the elderly people with (99.35%) and without anemia (98.33%). It is noteworthy that anemia was associated with LFHL only in the elderly population. The prevalence of LFHL in elderly people with anemia is 87.30%, which is higher than 80.71% in those without anemia. Based on our study results, we should particularly check and correct the hemoglobin level of elderly patients in routine clinical care to prevent the chronic progressive development of LFHL.

### Limitations

This study had several limitations. First, all data in NHANES are cross-sectional, and thus this study cannot establish the causal relationship between anemia and hearing threshold shift. Second, we cannot assess environmental, occupational, or recreational noises around the participants included in this study. Previous reports have highlighted the significance of noise-induced HL from both work and recreational activities [26,27]. Third, this study also could not evaluate the use of medications among these participants. Some drugs, such as gentamicin, sildenafil, and cisplatin chemotherapy, can damage the inner ear [28,29,30]. In addition, high doses of aspirin, other non-steroidal anti-inflammatory drugs, antimalarial drugs, or loop diuretics have also been reported to cause temporary tinnitus or HL [31]. Fourth, information about ear infection and abnormal bone growths or tumors in the outer or middle ear that can also result in HL was not available in the NHANES. Fifth, LFHL may be caused by Meniere’s disease, genetic conditions, central lesions, low spinal fluid pressure, and lithium use. These confounders cannot be adjusted in the multiple regression analysis of this study. Sixth, LFHL is also often related to conductive hearing loss, but bone thresholds are not available from the NHANES dataset. Therefore, we could not exclude conductive LFHL in this study. Seventh, previous evidence indicated that both anemia and hearing loss are sometimes partly explained by genetic deviations. Genetic components of anemia and HL were not available for this study. In addition, we did not consider some chronic diseases, like kidney diseases, rheumatism and cancer, which could be the comorbidity of both anemia and HL. Finally, the study findings should be cautiously generalized to other ethnicities because all patients included in this study were US residents.

In conclusion, our study demonstrated that anemia was associated with LFHL in the elderly population. However, further larger epidemiologic studies need to be conducted to confirm the effects of anemia on auditory threshold shifts in different ethnic groups and countries.

## Figures and Tables

**Table 1:** Demographic characteristics and comorbidities of subjects with anemia and the controls without anemia (*n* = 9131).

Variable	Subjects with Anemia (*n* = 918)		Subjects without Anemia (*n* = 8213)		*p* Value
	No.	%	No.	%	
Age Group (Years)					<0.001
≤19	313	34.1	3675	44.8	
20–29	72	7.8	721	8.8	
30–39	73	8.0	664	8.1	
40–49	85	9.3	629	7.7	
50–59	68	7.4	673	8.2	
60–69	74	8.1	610	7.4	
≥70	233	25.4	1241	15.1	
Sex					<0.001
Male	367	40.0	4292	52.3	
Female	551	60.0	3921	47.7	
Race					<0.001
Non-Hispanic white	224	24.4	3285	40.0	
Non-Hispanic black	446	48.6	1871	22.8	
Other	248	27.0	3057	37.2	
Ever had diagnosis					
Hypertension	329	35.8	1835	22.3	<0.001
Diabetes	128	13.9	570	6.9	<0.001
Coronary heart disease	44	4.8	212	2.6	<0.001
Heart failure	42	4.6	154	1.9	<0.001
Stroke	49	5.3	178	2.2	<0.001

**Table 2:** High-PTA, low-PTA, and prevalence of hearing loss in subjects with and without anemia.

Variables	Total (*n* = 9131)		Subjects with Anemia (*n* = 918)		Subjects without Anemia (*n* = 8213)		*p* Value
	*n*, %		*n*, %		*n*, %		
Low-PTA (dB)							
Right Ear	11.98 ± 12.58		15.04 ± 14.45		11.64 ± 12.31		<0.001
Left Ear	11.97 ± 12.71		15.57 ± 15.37		11.56 ± 12.31		<0.001
High-PTA (dB)							
Right Ear	19.08 ± 20.74		23.82 ± 23.06		18.55 ± 20.39		<0.001
Left Ear	20.04 ± 21.35		24.71 ± 23.80		19.51 ± 20.99		<0.001
Hearing Loss							
HL	5073	55.56	589	64.16	4484	54.60	<0.001
LFHL	3073	33.65	391	42.59	2682	32.66	<0.001
HFHL	4825	52.84	564	61.44	4261	51.88	<0.001

Low-PTA, pure tone average at low frequencies; High-PTA, pure tone average at high frequencies; LFHL, low-frequency hearing loss; HFHL, high-frequency hearing loss; HL, hearing loss.

**Table 3:** High-PTA, low-PTA, and prevalence of hearing loss in subjects with and without anemia according to the age group.

Variables	Elderly Population (≥60 Years Old)			Adult Population (20–59 Years Old)			Adolescent Population (≤19 Years Old)		
	Subjects with Anemia (*n* = 307)	Subjects without Anemia (*n* = 1851)	*p* Value	Subjects with Anemia (*n* = 298)	Subjects without Anemia (*n* = 2687)	*p* Value	Subjects with Anemia (*n* = 313)	Subjects without Anemia (*n* = 3675)	*p* Value
Low-PTA (dB)									
Right Ear	28.07 ± 15.23	25.13 ± 15.32	0.002	9.93 ± 8.98	9.24 ± 9.15	0.213	7.14 ± 7.41	6.60 ± 6.15	0.212
Left Ear	29.25 ± 16.49	25.01 ± 15.30	<0.001	10.55 ± 10.09	9.21 ± 8.84	0.028	6.93 ± 6.90	6.51 ± 6.57	0.278
High-PTA (dB)									
Right Ear	48.20 ± 20.40	45.22 ± 21.57	0.024	15.44 ± 13.56	15.82 ± 14.30	0.231	7.88 ± 8.55	7.12 ± 7.16	0.129
Left Ear	50.54 ± 20.50	47.00 ± 21.52	0.007	15.79 ± 13.05	16.98 ± 14.85	0.140	7.87 ± 8.57	7.52 ± 7.83	0.490
Hearing Loss									
HL	306 (99.67)	1825 (98.60)	0.163	185 (62.08)	1655 (61.59)	0.870	98 (31.31)	1004 (27.32)	0.130
LFHL	268 (87.30)	1494 (80.71)	0.006	80 (26.85)	717 (26.68)	0.952	43 (13.74)	471 (12.82)	0.640
HFHL	305 (99.35)	1820 (98.33)	0.216	178 (59.73)	1606 (59.77)	0.990	81 (25.88)	835 (22.72)	0.202

Note: Low-PTA, pure tone average at low frequencies; High-PTA, pure tone average at high frequencies; LFHL, low-frequency hearing loss; HFHL, high-frequency hearing loss; HL, hearing loss.

**Table 4:** Regression analyses of relationships between pure tone average and anemia according to different age groups.

Models	Variables	High-PTA (dB)				Low-PTA (dB)			
		Right Ear		Left Ear		Right Ear		Left Ear	
		β	*p* Value	β	*p* Value	β	*p* Value	β	*p* Value
Model 1 ^a^	All Patients with Anemia	5.27	<0.001	5.20	<0.001	3.40	<0.001	4.01	<0.001
	Elderly Patients with Anemia	2.99	0.024	3.54	0.007	2.93	0.002	4.24	<0.001
	Adult Patients with Anemia	−0.38	0.662	−1.20	0.182	0.69	0.213	1.34	0.015
	Adolescent Patients with Anemia	0.76	0.077	0.35	0.455	0.54	0.144	0.42	0.278
Model 2 ^b^	All Patients with Anemia ^c^	2.35	<0.001	2.33	<0.001	1.53	<0.001	2.25	<0.001
	Elderly Patients with Anemia ^d^	3.76	0.002	4.03	0.001	3.55	<0.001	4.64	<0.001
	Adult Patients with Anemia ^d^	1.42	0.102	1.42	0.109	0.55	0.340	1.37	0.015
	Adolescent Patients with Anemia ^e^	0.79	0.073	0.59	0.210	0.65	0.085	0.77	0.053

Note: Low-PTA, pure tone average at low frequencies; High-PTA, pure tone average at high frequencies.^a^ Model 1: Univariate regression; ^b^ Model 2: Multiple regression; ^c^ Adjusted covariates: sex, race, age, hypertension, diabetes, coronary heart disease, heart failure, stroke; ^d^ Adjusted covariates: sex, race, hypertension, diabetes, coronary heart disease, heart failure, stroke; ^e^ Adjusted covariates: sex and race.

## Supplementary Materials

The following are available online at https://www.mdpi.com/1660-4601/17/11/3916/s1, Table S1: Regression analyses of relationships between pure tone average and anemia according to sex and ethnicities.
