# Supplementary material for: Association between Anemia and Auditory Threshold Shifts in the US Population: National Health and Nutrition Examination Survey
Source: Int J Environ Res Public Health. 2020 Jun 1;17(11):3916. doi: 10.3390/ijerph17113916 (PMC7312977; doi:10.3390/ijerph17113916)
Supplement: Supplementary file 1 [file ijerph-17-03916-s001.docx]

**Supplementary Table 1.** Regression analyses of relationships between pure tone average and anemia according to sex and ethnicities.

| **Models** | Variables | High-PTA (dB) | | | | Low-PTA (dB) | | | |
| --- | --- | --- | --- | --- | --- | --- | --- | --- | --- |
|  |  | Right Ear | | Left Ear | | Right Ear | | Left Ear | |
|  |  | β | *p* value | β | *p* value | β | *p* value | β | *p* value |
| Model 1^a^ | Women with Anemia | 1.54 | .058 | 2.02 | .016 | 1.52 | .007 | 2.52 | < .001 |
|  | Men with Anemia | 12.05 | < .001 | 11.54 | < .001 | 6.10 | < .001 | 6.36 | < .001 |
|  | Non-Hispanic White with Anemia | 16.92 | < .001 | 16.85 | < .001 | 8.88 | < .001 | 9.99 | < .001 |
|  | Non-Hispanic Black with Anemia | 3.19 | < .001 | 3.32 | < .001 | 2.00 | < .001 | 2.32 | < .001 |
|  | Other Ethnicities with Anemia | 4.52 | < .001 | 4.58 | < .001 | 3.69 | < .001 | 4.54 | < .001 |
| Model 2^b,c^ | Women with Anemia | 1.29 | .017 | 1.77 | .001 | 1.22 | .006 | 2.32 | < .001 |
|  | Men with Anemia | 2.52 | < .001 | 1.60 | .033 | 1.94 | < .001 | 1.98 | < .001 |
|  | Non-Hispanic White with Anemia | 3.41 | < .001 | 3.04 | .002 | 1.98 | .012 | 2.98 | < .001 |
|  | Non-Hispanic Black with Anemia | 1.75 | .004 | 1.83 | .003 | 0.89 | .064 | 1.30 | .006 |
|  | Other Ethnicities with Anemia | 1.04 | .167 | 1.00 | .199 | 1.71 | .002 | 2.60 | < .001 |

Note: Low-PTA = Pure tone average at low frequencies; High-PTA = Pure tone average at high frequencies. ^a^ Model 1: Univariate regression; ^b^ Model 2: Multiple regression; ^c^ Adjusted covariates: patients’ demographics, hypertension, diabetes, coronary heart disease, heart failure, stroke.
